# *In Vitro* Synergistic Effect of Curcumin in Combination with Third Generation Cephalosporins against Bacteria Associated with Infectious Diarrhea

**DOI:** 10.1155/2014/561456

**Published:** 2014-05-18

**Authors:** Nishanth Kumar Sasidharan, Sreerag Ravikumar Sreekala, Jubi Jacob, Bala Nambisan

**Affiliations:** ^1^Agroprocessing and Natural Products Division, National Institute for Interdisciplinary Science and Technology (NIIST), Council of Scientific and Industrial Research (CSIR), Thiruvananthapuram, Kerala 695 019, India; ^2^Division of Crop Protection and Division of Crop Utilization, Central Tuber Crops Research Institute, Sreekariyam, Thiruvananthapuram 695017, India

## Abstract

Diarrhea is one of the leading causes of morbidity and mortality in humans in developed and developing countries. Furthermore, increased resistance to antibiotics has resulted in serious challenges in the treatment of this infectious disease worldwide. Therefore, there exists a need to develop alternative natural or combination drug therapies. The aim of the present study was to investigate the synergistic effect of curcumin-1 in combination with three antibiotics against five diarrhea causing bacteria. The antibacterial activity of curcumin-1 and antibiotics was assessed by the broth microdilution method, checkerboard dilution test, and time-kill assay. Antimicrobial activity of curcumin-1 was observed against all tested strains. The MICs of curcumin-1 against test bacteria ranged from 125 to 1000 **μ**g/mL. In the checkerboard test, curcumin-1 markedly reduced the MICs of the antibiotics cefaclor, cefodizime, and cefotaxime. Significant synergistic effect was recorded by curcumin-1 in combination with cefotaxime. The toxicity of curcumin-1 with and without antibiotics was tested against foreskin (FS) normal fibroblast and no significant cytotoxicity was observed. From our result it is evident that curcumin-1 enhances the antibiotic potentials against diarrhea causing bacteria in *in vitro* condition. This study suggested that curcumin-1 in combination with antibiotics could lead to the development of new combination of antibiotics against diarrhea causing bacteria.

## 1. Introduction

The widespread emergence of resistance to multiple antimicrobial agents in pathogenic bacteria has become a significant global public health threat. Multidrug-resistant bacterial infections cause significant patient mortality and morbidity, and rising antibiotic resistance is a serious threat to the vast medical achievements made possible by antibiotics over the past 70 years [1]. Nowhere is the concept of antimicrobial resistance better portrayed than with the gram-negative bacilli, which have proven to be tough adversaries for clinicians and researchers alike [2]. Resistance to the current library of antibacterial drugs is a serious problem in all parts of the world including the Asia-Pacific region, Latin America, Europe, and North America.

The drug combinations are one such approach to fight against the multidrug-resistant bacteria (MDR) effectively. Such efforts include antibiotic-antibiotic combinations and the pairing of an antibiotic with a nonantibiotic adjuvant molecule. Antibiotics-natural compound combinations were also used to compete against MDR bacteria [3]. Natural products offer an untold diversity of chemical structures. These natural compounds often serve as lead molecules whose activities can be enhanced by manipulation through combinations with chemicals and by synthetic chemistry [4].

Diarrhea is an important clinical problem [5] and a leading cause of morbidity and mortality in human beings worldwide [6]. Diarrhea is an intestinal infection responsible for death in the elderly in developed countries [7] and is responsible for the deaths of 3-4 million infants and young children each year worldwide [8]. It accounted for 1.78 million deaths in underdeveloped countries [9]. The important bacterial pathogens responsible for causing diarrhea are diarrheagenic* Escherichia coli, Salmonella typhi*,* Bacillus cereus*,* Campylobacter jejuni*,* Aeromonas hydrophila, Shigella* spp.*, Yersinia* spp., and* Vibrio cholera,* and the main causative parasites in contaminated domestic water supplies included* Giardia intestinalis* and* Cryptosporidium parvum* [10].

Curcumin [1,7-bis(4-hydroxy-3-methoxyphenyl)-1,6-heptadiene-3,5-dione] is a natural polyphenolic compound isolated from the rhizome of* Curcuma longa*. Curcumin (CUR-1) is commonly used as a spice and food coloring agent throughout Asia. CUR-1 is also used for the treatment of variety of diseases ranging from acute infection to chronic disease (e.g., inflammatory bowel syndrome, diabetes, and asthma) [11, 12]. CUR-1 has also been found to possess many beneficial biological activities, including antioxidant, antimicrobial, antitumor, and anti-inflammatory properties as well as a potent inhibitory effect on nuclear factor-kappa B [13–16].

In the present study, we investigated the synergistic activity of CUR-1 with three clinically used third generation cephalosporin antibiotics (cefaclor, cefodizime, and cefotaxime) against bacteria associated with diarrhea.

## 2. Materials and Methods

### 2.1. Extraction and Isolation of CUR-I

The rhizomes of* Curcuma longa* were obtained from the Central Tuber Crops Research Institute (CTCRI) field. The rhizomes were dried at room temperature (30–35°C), pulverized, and stored at 8°C for further studies. The powdered rhizomes (100 g) were first defatted with 1000 mL hexane in a Soxhlet apparatus for 5 h and then extracted with 1000 mL chloroform for 4 h. The chloroform layer was filtered and evaporated under vacuum (40°C) to produce a crude curcuminoid-rich extract (5.21 g). The crude extract (5.2 g) was loaded on a silica gel column (200 g, 60–120 mesh, 5 × 60 cm glass) and successively eluted with hexane 500 mL, chloroform: hexane (20–80%, 1000 mL each), chloroform 1000 mL, and acetone-chloroform (1–5%, 1000 mL each). The fractions were collected and spotted on thin-layer chromatography (TLC) sheets coated with silica gel. Fractions that showed the same pattern on TLC (100 mL each) were pooled [CUR-I (fractions 35–56), CUR-II (fractions 60–65), and CUR-III (fractions 72–76) were obtained successively] and the solvent was removed to obtain the powder.

### 2.2. High-Pressure Liquid Chromatography (HPLC)

The purity of CUR-I, CUR-II, and CUR-III was analyzed by HPLC in a Shimadzu LC-10AT liquid chromatography system (LC; Shimadzu, Singapore) with SPD-AuV detector. 20 *μ*L of sample was injected and the elution was carried out with gradient solvent systems with a flow rate of 1 mL/min at ambient temperature. Column used was C18 (250 × 4.6 mm). The mobile phase consisted of methanol (A), 2% acetic acid (B), and acetonitrile (C) and was programmed linearly from 45 to 65% acetonitrile in B for 0–15 min. The gradient then went from 65 to 45% acetonitrile in B for 15–20 min, with a constant of 5% A, and was measured at 420 nm.

**Figure 1:**
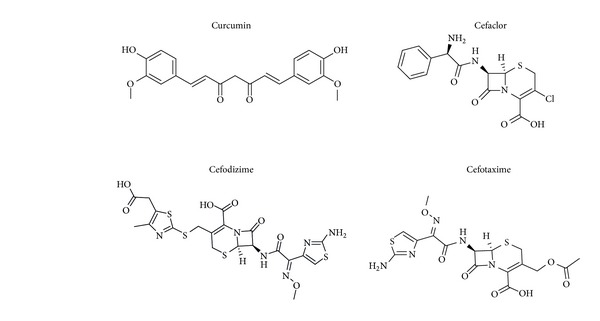
Structure of CUR-1 and antibiotics used in the present study. CUR-1 was isolated and purified from* Curcuma longa* and antibiotics were purchased from Sigma-Aldrich.

The purity of CUR-I, CUR-II, and CUR-III was found to be 98.9%, 98%, and 97.1%, respectively (data not shown). CUR-I, CUR-II, and CUR-III showed single peaks at retention times of 11.13, 13.49, and 14.56 min, respectively. The identity of each peak was confirmed by determination of retention times and by comparing with standards. CUR-I formed as needle shaped bright yellow crystals, CUR-I1 as light yellow crystals, CUR-I11 as reddish orange color crystals. CUR-I was the major component (<83%) of the crude extract which was used in the present study (Figure 1).

### 2.3. Antibiotics and Media

The standard antibiotics cefaclor, cefodizime, and cefotaxime (Figure 1) were purchased from Sigma-Aldrich, USA. Microbiological media were from Hi-Media Laboratories Limited, Mumbai, India.

### 2.4. Bacterial Strains and Growth Conditions

The bacteria used in this study included* Staphylococcus aureus* MTCC 902,* Bacillus subtilis* MTCC 2756,* Escherichia coli* MTCC 2622,* Pseudomonas aeruginosa* MTCC 2642, and* Vibrio cholerae* MTCC 3905. All the test microorganisms were purchased from Microbial Type Culture Collection Centre, IMTECH, Chandigarh, India. The test bacteria were maintained on nutrient agar slants.

### 2.5. Antimicrobial Activity of CUR and Antibiotics

#### 2.5.1. Minimal Inhibitory Concentration (MIC)

The minimal inhibitory concentration (MIC) test of CUR-I and antibiotics was recorded by using the microdilution broth method with some modifications in CLSI [17]. Serial 2-fold dilutions of CUR-I, cefaclor, cefodizime, and cefotaxime were prepared in 96-well sterile microplates containing Mueller Hinton broth (MHB). Ten microliters of the test bacterial suspension was inoculated in each well to give a final concentration of 10^4^ CFU. CUR-I and antibiotics ranged from 1024 to 0.5 *μ*g/mL were used for testing. The inhibition of growth was demonstrated by optical density at 600 nm using a microplate reader (Bio-Rad, USA) after 24 h incubation at 35°C. Considering the total growth (100%) in the control well (MHB + bacteria), the percentage of growth reduction was attributed to the remaining wells. Control solution containing dimethyl sulfoxide and sterile water which is used for dissolving CUR-1 and antibiotics, respectively, was included in this experiment to exclude the possibility of toxic effects on the microorganisms. The MIC was reported as the lowest concentration of CUR-I and antibiotics that inhibited the bacterial growth after 24 h of incubation at 37°C.

#### 2.5.2. Minimal Bactericidal Concentration (MBC)

MBC was recorded as a lowest concentration of CUR-1 and antibiotics that kill 99.9% of the bacterial inocula after 24 h incubation at 37°C. Each experiment was repeated at least three times. MBC values were determined by removing 100 *μ*L of bacterial suspension from culture demonstrating no visible growth in MIC experiment and inoculating in nutrient agar plates. Plates were incubated at 37°C for a total period of 24 h. The MBC is determined with the wells whose concentrations are greater than MIC [18].

### 2.6. Determination of the* In Vitro* Effects of Combinations of CUR-I and Antibiotics

The antimicrobial effects of different combinations of 2 or more antimicrobial agents were assessed using the checkerboard test [19]. Checkerboard synergy testing is among the most widely used standard techniques to determine the synergistic activity of antibiotic combinations. It is based on microdilution susceptibility testing of antibiotic combinations. The antimicrobial assays were performed with CUR-I in combination with cefaclor, cefodizime, and cefotaxime. Serial dilutions of CUR-I with these antibiotics were mixed in 1 : 1 ratio in MHB. The inocula were prepared from colonies that had been grown on Mueller Hinton agar (MHA) overnight. The final bacterial concentration after inoculation was 2 × 10^5^ CFU/mL. The MIC was determined after 24 h incubation at 37°C. The MIC was defined as the lowest concentration of CUR-I, alone or in combination with antibiotics, visibly inhibiting the growth of bacteria by measuring the OD at 600 nm using a microplate reader (Bio-Rad, USA). Each experiment was repeated thrice. The* in vitro* interaction between the CUR-I and antibiotics was quantified by determining the fractional inhibitory concentration (FIC). The FIC index (FICI) was calculated using the following formula:

(1) $$\begin{array}{c} \text{FIC}\;\text{index}=\text{FICA}+\text{FICB}=\frac{\left[\text{A}\right]}{\text{MICA}}+\frac{\left[\text{B}\right]}{\text{MICB}}, \end{array}$$

where [A] is the concentration of drug A and MICA and FICA are the MIC and the FIC of drug A for the organism, respectively, whereas [B], MICB, and FICB are similarly defined for drug B. The FIC index obtained was interpreted as follows: <0.5 denotes synergy; 0.5–0.75 denotes partial synergy; 0.76–1 denotes an additive effect; 1–4 denotes indifference; and >4 denotes antagonism [20].

### 2.7. The Time-Kill Assay

The time-kill assay, in order to study the synergistic effects of CUR-I + antibiotic on bacterial growth by the seven time intervals (0, 2, 4, 6, 12, 24, and 48 h), was performed as described by Chang et al. [19]. Bacterial cultures incubated in MHA for 24 h at 37°C were diluted with fresh MHB to approximately 2 × 10^5^ CFU/mL and the diluted cultures were preincubated at 37°C for 24 h. Aliquots (0.1 mL) of the culture were removed at seven time intervals (0, 2, 4, 6, 12, 24, and 48 h) of incubation, and serial 10-fold dilutions were prepared in normal saline as needed. The numbers of viable cells were determined on MHA plate after 24 h incubation. Colony counts were performed on plates, and 20–400 colonies were enumerated. The lower limit of sensitivity of colony counts was 100 CFU/mL. The antimicrobial agents used were considered bactericidal at the lowest concentration that reduced the original inoculum by 3 log10 CFU/mL (99.9%) for each of the indicated times. On the other hand, they were considered bacteriostatic if the inoculum was reduced by 0–3 log10 CFU/mL. The time-kill assays for all experiments were performed at least thrice for confirmation of the results; the data are represented as mean ± standard deviation.

### 2.8. Human Normal Cell Toxicity Determination

Foreskin normal fibroblast (FS) cells were used to evaluate the toxicity effect of CUR-1 with and without cefaclor, cefodizime, and cefotaxime using the method described by Zhang et al. [21], with a slight modification. 3-(4,5-Dimethylthiazol-2-yl)-2,5-diphenyltetrazoliumbromide (MTT, a tetrazole) assay (Sigma-Aldrich, St. Louis, MO) was used to determine the relative cell viability, according to the manufacturer's instruction.

Briefly, FS cells were cultured in RPMI medium 1640, supplemented with 10% fetal bovine serum (Thermo Scientific, Lafayette, CO), 100 U/mL streptomycin, 100 mg/mL penicillin, 4 mM L-glutamine, 1% nonessential amino acids, and 1 mM sodium pyruvate. Cells were maintained at 37°C and 5% CO_2_ in a humidified incubator. From the top to the bottom of 96-well microtiter plate there was a series of twofold dilutions of the compounds: CUR-1 alone in columns 1 and 2, cefaclor alone in columns 3 and 4, cefodizime B alone in columns 5 and 6, cefotaxime B alone in columns 7 and 8, CUR-1 in combination with cefaclor in columns 9 and 10, CUR-1 in combination with cefodizime in columns 11 and 12, and CUR-1 in combination with cefotaxime in columns 13 and 14. Wells in columns 15 and 16 were treated as controls (cells not exposed to antimicrobial agent). Concentrations used were 8 *μ*g/mL cefodizime, 16 *μ*g/mL cefodizime or cefotaxime 8 *μ*g/mL alone and in combination with 1000 *μ*g/mL of CUR-1, and 1000 *μ*g/mL CUR-1 alone. The same amount of FS cells was seeded in each of the selected wells and incubated for 24 h at 37°C CO_2_ incubator with a humidified chamber. A 30 mL solution of MTT (5 mg/mL in phosphate-buffered saline) was added in each of the selected wells and incubated for 2 h at 37°C. Relative cell viability was determined at 595 nm with microplate reader (Bio-Rad, USA). The experiments were performed four times with duplicate wells for each experiment.

## 3. Results

### 3.1. Antimicrobial Activity of CUR and Antibiotics

Antimicrobial susceptibility tests of CUR-I and antibiotics against five diarrhea causing bacteria were performed using the standard broth microdilution method. The CUR-I and antibiotics showed antimicrobial activity against all the tested strains and the result were shown in Table 1. The MIC/MBC values of CUR-I against the test bacterial strains ranged from 125 to 1000 *μ*g/mL. The MIC/MBC of cefaclor ranged from 2 to 16 *μ*g/mL, cefodizime ranged from 2 to 32 *μ*g/mL, and cefotaxime ranged from 2 to 16 *μ*g/mL.

### 3.2. Evaluation of Synergistic Effect

The combined effects of CUR-1 and three antibiotics were tested on five diarrhea causing bacteria. CUR-1 significantly lowered the MICs of antibiotics (cefaclor, cefodizime, and cefotaxime) against the test bacteria. The synergistic effects of CUR-1 and three antibiotics combination are shown in Table 2. Significant synergistic effect was recorded by CUR-1 in combination with cefotaxime and FICIs for this combination range from 0.03 to 0.14 against the test bacteria. All of the organisms examined exhibited a 3- to 8-fold reduction in MIC values with CUR-1 and the three antibiotics. These results showed that CUR-1 in combination with antibiotics could effectively inhibit diarrhea causing bacteria.

### 3.3. Time-Kill Assay

**Figure 2:**
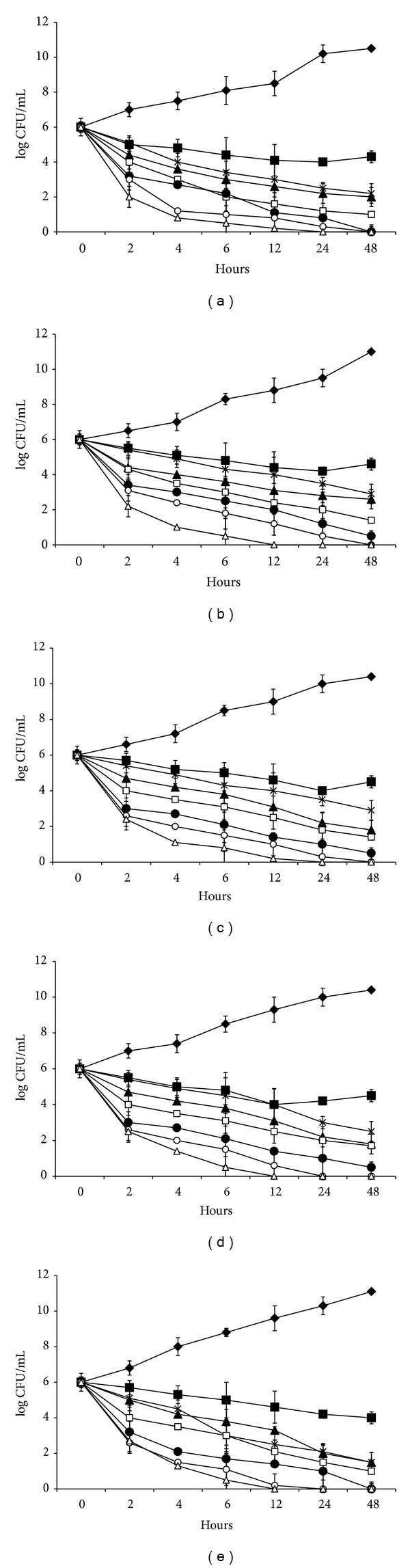
Time-kill cure of CUR-1 and antibiotics alone and in combination against bacteria. The strains at a starting inoculum density of 10^6^ CFU/mL were used. At 0, 2, 4, 6, 8, 12, 24, and 48 h aliquots were removed from each test tube to examine the cell viability. The experiments were performed three times. Data are expressed as mean ± standard deviation. (a)* Staphylococcus aureus*, (b)* Bacillus subtilis*, (c)* Escherichia coli* MTCC 2622, (d)* Pseudomonas aeruginosa*, and (e)* Vibrio cholerae.* **—** *◆* **—**: control,** —**■**—**: CUR-1,** —**▲**—**: cefaclor,** —×—**: cefodizime,** —**□**—**: cefotaxime,** —**●**—**: CUR-1+cefaclor,** —**○**—**: CUR-1+cefodizime,** —**▵**—**: CUR-1+cefotaxime.

To confirm the synergistic effects of CUR-1 and antibiotics against test bacteria, a time-kill assay was performed and the results were shown in Figure 2. CUR-1 in combination with antibiotics showed a concentration-dependent activity, resulting in significant reduction of the CFUs. From the result it is clear that CUR-1 alone did not record cell reduction even after 24 h. But antibiotics alone recorded 3 log_10_-fold reductions in the bacterial count between 6 to 12 h. The combination of CUR-1 and antibiotics recorded 3 log_10_-fold reductions in the bacterial count after 2 h and a complete reduction in the colony count was recorded after 12 h. Similar to checkerboard assay, CUR-1 in combination with cefotaxime recorded significant reduction in the colony count (Figure 2). Regrowth of test bacteria was observed for CUR-1 whereas it is not recorded in the combination of CUR-1 and antibiotics.

### 3.4. Toxicity Study

**Figure 3:**
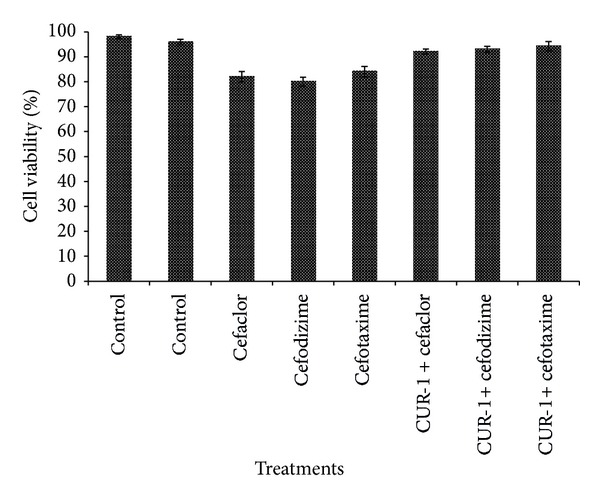
Nontoxicity effect of CUR-1 and antibiotics alone and in combination with human foreskin (FS) cells. Cells were cultured without antibiotics and curcumin was used as control. Data are expressed as % control and each column represents the mean ± SD of three independent experiments.

To evaluate CUR-1 with and without antibiotics that may be toxic to human cell, we used FS normal fibroblast cell line as a substitute system to impersonate potential therapeutic side effects in human body. The result showed that there was no significant cytotoxicity in CUR-1 alone and in combination with antibiotics. Cell viability in CUR-1 alone and in combination with antibiotics tested wells was greater than 95% compared with that of the control (Figure 3). But fixed concentration of antibiotics alone recorded slight toxicity (Figure 3). These results indicated that CUR-1 with and without antibiotics is not toxic to the tested human cell line. The results of* in vitro* study clearly indicated that CUR-1 in combination with antibiotics may be safe for the treatment of pathogenic bacteria.

## 4. Discussion

MDR bacteria represent an enormous challenge to modern health care systems. Although some new agents have been introduced in the last 10 years (e.g., linezolid, daptomycin, and tigecycline) [22, 23], the widespread emergence of bacterial resistance to a large number of antimicrobial agents poses major health problems because of difficulties in treatment [23]. The indiscriminate use of antimicrobial agents in the treatment of bacterial infections has led to the emergence and spread of resistant strains, and it resulted in a great loss of clinical efficacy of previously effective first-line antimicrobials which results in shifting of antimicrobial treatment regimen to second-line or third-line antimicrobial agents that are often more expensive with many side effects [24].

The erosion of effective treatments by resistance, combined with a drug development pipeline that is almost dry, has transformed interest in using unconventional therapies for bacterial infections. Synergism of natural products and antimicrobial agents is a thrust area of phytomedicinal research, developing novel perspective of phytopharmaceuticals. The synergism of plant-derived compounds and antimicrobial agents has been evaluated previously against pathogenic microorganisms [25, 26].

In this study we looked at the effects of combining the curcumin with three antibiotics against bacteria. In the present study curcumin alone recorded antimicrobial activity at higher concentration. This is in accordance with the previous report [27]. But curcumin in combination with antibiotics recorded significant synergistic effect. When used together, the drugs were not only synergistic but also bactericidal and prevented the regrowth of bacteria in time-kill assays. These data suggest that curcumin in combination with antibiotics could be a useful option for the treatment of complicated bacterial infections. In addition to achieving these synergistic effects, the combinations of two or more compounds are essential for the following reasons: (1) to prevent or suppress the emergence of resistant strains, (2) to decrease dose-related toxicity, as a result dosage, and (3) to attain a broad spectrum of activity [28].

Bacteria develop resistance to antibiotics and this is associated with an increase in the MIC of one or more antibiotics. This means that when a patient has a clinical disease that is caused by a resistant bacterium, treatment with the antibiotic to which the bacterium is resistant is less effective. Sometimes, it is possible to simply increase the dose of the antibiotic to overcome the bacterial resistance. However, for many antibiotics, such as aminoglycosides, it is not feasible to significantly increase the dose of the antibiotic because of toxic side effects. In these circumstances, benefit for the patient could be achieved by enhancing the effect of the antibiotic against resistant bacteria. Here, we clearly showed that curcumin enhances the activity of cefaclor, cefodizime, and cefotaxime. Most interestingly, the bactericidal activities of the tested drugs were significantly enhanced. Synergistic effect of curcumin with antibiotics is previously reported against methicillin-resistant* Staphylococcus aureus* and* Candida albicans* [29, 30]. Synergistic effect of curcumin and antibiotics against bacteria associated with diarrhea is reported here for the first time.

Recently some edible natural products and food ingredients have been reported to enhance the antibacterial activity of different antibiotics such as nitrofurantoin and clindamycin [31–33]. As mentioned above the different investigation has been carried out on the biological activities of curcumin but the combination effects of this natural product with different antibiotics have not been demonstrated. In this study we also investigated the effect of curcumin on human normal foreskin (FS) cell line and curcumin has no cytotoxicity to normal human cells. Curcumin has been reported having an extremely good safety profile and no toxicity observed when taken at doses as high as 12 g/day in* in vivo* test [34, 35]. In our study also curcumin recorded no toxicity up to 200 *μ*g/mL.

At this time the reason for these enchantments and the reason for these differences are not known and merit investigation. Efflux transporter mediated bacterial resistance to different antibiotics [36] and curcumin may inhibit this efflux pump system. This is the first report of combination effect of curcumin derived from* Curcuma longa* with different antibiotics. Today, curcumin as a food ingredient has drawn the attention of many scientists because of its extensive pharmaceutical properties [34, 35].

## 5. Conclusion

In conclusion, our results indicate that a combination of CUR-1 and antibiotics exhibited good synergism against bacteria associated with diarrhea. The result confirmed that curcumin as a safe natural compound could also serve as valuable probes to study the structure-function relationships of the antibiotic resistance reversal agents. Therefore, this compound has a good potential for combination therapy against bacteria. Moreover our result also indicated that curcumin enhanced the potential of antibiotics in* in vitro* condition. This new finding of combination treatment with CUR-1 and antibiotics might provide an alternative approach to overcome antibacterial drug resistance. However, further study is necessary to determine the underlying mechanism of this synergistic action. Moreover further* in vivo* and clinical studies will be required to support this suggestion.

## Figures and Tables

**Table 1:** Antibacterial activity of CUR-I and antibiotics against bacteria.

Test compounds	MIC/MBC (*μ*g/mL)				
	*S. aureus *	*B. subtilis *	*E. coli *	*P. aeruginosa *	*V. cholerae *
CUR-I	250/500	250/500	500/500	500/1000	125/250
Cefaclor	8/16	8/16	4/8	8/8	2/4
Cefodizime	16/32	16/16	2/4	8/16	4/4
Cefotaxime	4/8	4/4	8/16	2/4	4/4

Values represent mean of three replications.

**Table 2:** Synergistic effects of the CUR-I with antibiotics against bacteria.

Test bacteria	Agent	MIC/MBC (*μ*g/mL)		FIC/FBC	FICI^2^/FBCI^3^	Outcome
		Alone	Combination^1^			
* S. aureus *	CUR-I Cefaclor	250/500 8/16	8/16 1/2	0.03/0.03 0.06/0.13	0.09/0.16	Synergistic/synergistic
	CUR-I Cefodizime	250/500 16/32	16/32 1/1	0.06/0.06 0.06/0.03	0.12/0.09	Synergistic/synergistic
	CUR-I Cefotaxime	250/500 4/8	4/8 0.12/0.25	0.01/0.01 0.03/0.03	0.04/0.04	Synergistic/synergistic

* B. subtilis *	CUR-I Cefaclor	250/500 8/16	32/64 2/4	0.13/0.13 0.13/0.13	0.26/0.26	Synergistic/synergistic
	CUR-I Cefodizime	250/500 16/16	64/64 2/4	0.26/0.13 0.13/0.25	0.39/0.38	Synergistic/synergistic
	CUR-I Cefotaxime	250/500 4/4	4/8 0.25/0.5	0.02/0.02 0.06/0.13	0.08/0.15	Synergistic/synergistic

* E. coli *	CUR-I Cefaclor	500/500 4/8	16/32 1/1	0.03/0.06 0.25/0.13	0.28/0.19	Synergistic/synergistic
	CUR-I Cefodizime	500/500 2/4	32/32 0.5/1	0.06/0.06 0.25/0.25	0.31/0.31	Synergistic/synergistic
	CUR-I Cefotaxime	500/500 8/16	8/8 0.12/0.12	0.02/0.02 0.01/0.01	**0.03/0.03**	Synergistic/synergistic

* P. aeruginosa *	CUR-I Cefaclor	500/1000 8/8	64/125 2/2	0.13/0.13 0.25/0.25	0.38/0.38	Synergistic/synergistic
	CUR-I Cefodizime	500/1000 8/16	64/64 1/2	0.13/0.06 0.13/0.13	0.26/0.19	Synergistic/synergistic
	CUR-I Cefotaxime	500/1000 2/4	8/16 0.25/0.5	0.01/0.01 0.13/0.13	0.14/0.14	Synergistic/synergistic

* V. cholerae *	CUR-I Cefaclor	125/250 2/4	8/16 0.5/0.5	0.06/0.06 0.25/0.13	0.31/0.19	Synergistic/synergistic
	CUR-I Cefodizime	125/250 4/4	8/8 1/1	0.06/0.03 0.25/0.25	0.31/0.28	Synergistic/synergistic
	CUR-I Cefotaxime	125/250 4/4	2/4 0.12/0.25	0.02/0.02 0.03/0.06	0.05/0.08	Synergistic/synergistic

^1^The MIC and MBC of CUR-1 with antibiotics.

^2^The fractional inhibitory concentration index (FIC index).

^3^The fractional bactericidal concentration index (FBC index).

Significant FICI/FBCI values are shown in bold.
